# Genetic diversity and phylogenetic relationships of tsetse flies of the *palpalis* group in Congo Brazzaville based on mitochondrial *cox*1 gene sequences

**DOI:** 10.1186/s13071-020-04120-3

**Published:** 2020-05-14

**Authors:** Abraham Mayoke, Shadrack M. Muya, Rosemary Bateta, Paul O. Mireji, Sylvance O. Okoth, Samuel G. Onyoyo, Joanna E. Auma, Johnson O. Ouma

**Affiliations:** 1 Department of Molecular Biology and Biotechnology, Pan African University Institute for Basic Sciences, Technology & Innovation, PO Box 62000-00200, Nairobi, Kenya; 2 Kenya Agricultural and Livestock Research Organization, Biotechnology Research Institute, PO Box 362-00902, Kikuyu, Kenya; 3 Jomo Kenyatta University of Agriculture and Technology, Faculty of Biological Sciences, PO Box 62000-00200, Nairobi, Kenya; 4 African Technical Research Centre, Vector Health International, P.O. Box 15500, Arusha, Tanzania

**Keywords:** *Glossina palpalis palpalis*, *Glossina fuscipes*, Taxonomy, Genetic diversity, Cytochrome *c* oxidase, mtDNA, Phylogeny, Congo Brazzaville

## Abstract

**Background:**

Despite the morphological characterization established in the 1950s and 1960s, the identity of extant taxa that make up *Glossina fuscipes* (*s.l.*) in the Congo remains questionable. Previous claims of overlap between *G. fuscipes* (believed to be *G. f. quanzensis*) and *G. palpalis palpalis* around Brazzaville city further complicate the taxonomic status and population dynamics of the two taxa. This study aimed to determine the phylogenetic relationships between *G. fuscipes* (*s.l*.) and *G. p. palpalis* and to assess genetic variation among *G. fuscipes* (*s.l*.) populations in Congo Brazzaville.

**Methods:**

We collected 263 *G. fuscipes* (*s.l*.) from northern and central regions, and 65 *G. p. palpalis* from southern part of the country. The mitochondrial cytochrome *c* oxidase subunit 1 (*cox*1) gene was amplified using taxa-specific primer pairs. Sequence data were analyzed in DnaSP and Arlequin to assess the genetic diversity, differentiation and demographic history of *G. fuscipes* (*s.l*.) populations.

**Results:**

The general BLAST analysis yielded a similarity of 99% for *G. fuscipes* (*s.l.*) and *G. p. palpalis*. BLASTn analysis for *G. fuscipes* (*s.l*.) showed > 98% identity with GenBank sequences for *G. fuscipes* (*s.l*.), with BEMB population showing 100% similarity with *G. f. fuscipes*. *Glossina fuscipes* (*s.l*.) populations showed high haplotype diversity (H = 46, Hd = 0.884), moderate nucleotide diversity ( = 0.012) and moderate (F_ST_ = 0.072) to high (F_ST_ = 0.152) genetic differentiation. Most of the genetic variation (89.73%) was maintained within populations. The mismatch analysis and neutrality tests indicated recent tsetse population expansions.

**Conclusions:**

Phylogenetic analysis revealed minor differences between *G. fuscipes* (*s.l.*) and *G. p. palpalis.* Genetic diversity of *G. fuscipes* (*s.l.*) was high in the populations sampled except one. Genetic differentiation ranged from moderate to high among subpopulations. There was a restricted gene flow between *G. fuscipes* (*s.l.*) populations in the north and central part of the country. Genetic signatures based on *cox*1 showed recent expansion and recovery of *G. fuscipes* (*s.l.*) populations from previous bottlenecks. To fully understand the species distribution limits, we recommend further studies involving a wider sampling scheme including the swampy Mossaka focus for *G. fuscipes* (*s.l.*) and the entire range of *G. p. palpalis* in South Congo.
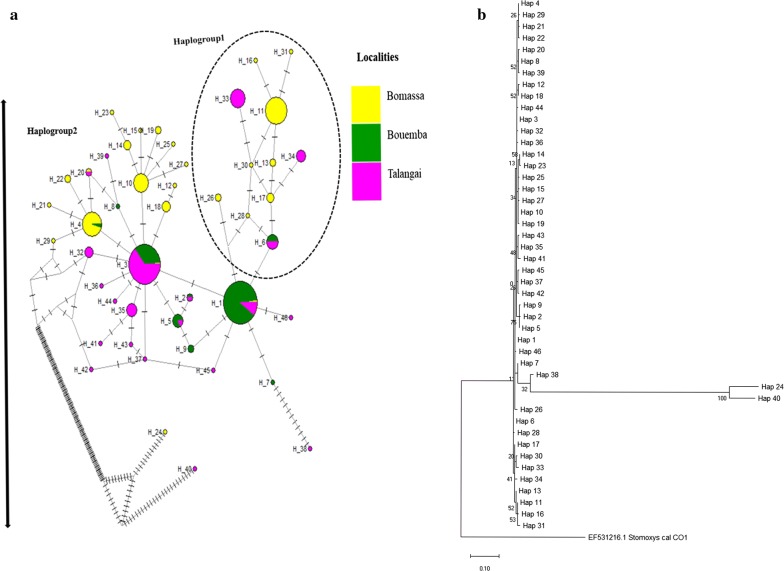

## Background

Riverine tsetse fly species are the most important vectors of human African trypanosomiasis (HAT) [1] in West and Central Africa where they mainly transmit *Trypanosoma brucei gambiense* [2]. These flies belong to the subgenus *Nemorhina* (*palpalis* group tsetse that typically inhabit lacustrine or riverine habitats) and can be broadly classified into two super-species i.e. *Glossina fuscipes* (*s.l.*) and *G. palpalis* (*s.l*.), which do coexist in certain situations. Apart from HAT, these vectors also transmit nagana, a debilitating disease that severely hinders livestock productivity in rural Africa [2, 3].

Based on morphological features, previous studies indicated that the two main tsetse vectors of HAT and nagana in Congo Brazzaville were *Glossina palpalis palpalis* occupying the southern part of the country up to the south of Brazzaville city around the Djoué River [4, 5], and *G. fuscipes quanzensis* [6] that extends its distribution from Gamboma to the Ngabe corridor along the Congo River banks [7]. The north of Congo was reported to be colonized by *Glossina fuscipes fuscipes* [8], with its distribution extending from Gamboma to the south of Cameroon [7]. Although *G. fuscipes* (*s.l.*) (including *G. f. quanzensis* and *G. f. fuscipes*) and *G. p. palpalis* are reported to be important vectors of HAT in Congo Brazzaville [8], there have been conflicting reports on their distribution limits in the country. Distinguishing *G. fuscipes* (*s.l*.) (including its subspecies above) and *G. palpalis* is very difficult as the morphological differences between the two species and the subspecies are very minor, and field workers have had to use species distribution maps to help them decide which species they are dealing with [9]. The identification of these tsetse species based on genitalia and other morphological features in Congo Brazzaville remains questionable [10], with different subspecies of *G. fuscipes* (*G. f. quanzensis*, *G. f. fuscipes* and *G. f. martinii*) being considered synonymous taxa. Whereas morphological traits (e.g. features of the genitalia) have been useful in differentiating tsetse taxa, such traits are variable and require highly skilled and rare *Glossina* taxonomists. Unfortunately, in Congo Brazzaville, there have been no attempts to resolve the taxonomy of the closely related *palpalis* group tsetse by molecular characterization. Therefore, to date, there is no information on the molecular taxonomic status and distribution limits of the various subspecies of *G. fuscipes* in Congo Brazzaville and how they relate phylogenetically with *G. p. palpalis* which is reported to infest the southern part of the country.

Previous reports indicated the coexistence of *G. p. palpalis* and *G. f. quanzensis* in south Congo up till the 1950s [3]. However, this was no longer the case in the 1960s as it was reported that *G. p. palpalis* had replaced *G. f. quanzensis* [10] due to competition, although it was later claimed that *G. f. quanzensis* reappeared in the south of Brazzaville [7]. In a separate study, it was demonstrated by using morphometric features that *G. f. quanzensis* collected from three different localities were not identical, and interestingly, no clinal variation in size was observed between *G. f. fuscipes* and *G. f. quanzensis* subspecies [4]. However, differences were attributed to their biotopes and locations of capture, e.g. *G. f. quanzensis* found in the Ngabe corridor was slightly smaller compared to *G. f. fuscipes* found in Mossaka [4]. Apart from the claimed replacement of *G. f. quanzensis* by *G. p. palpalis*, it was previously reported that there could be a hybrid zone of *G. p. palpalis* and *G. f. quanzensis* that existed around Brazzaville [5, 11]. The replacement phenomenon and the possible existence of a hybrid zone between these taxa around Brazzaville town call for the use of molecular genetic tools to better understand the taxonomy of the extant *palpalis* group tsetse flies in Congo Brazzaville.

Proper understanding of the taxonomic status and knowledge on population dynamics including population genetic diversity, gene flow and genetic differentiation is important for effective tsetse control, particularly by using area-wide genetic approaches such as the sterile insect technique (SIT) [12]. Mitochondrial DNA (mtDNA) which encodes 22 tRNA genes and 13 proteins including cytochrome oxidases, is a highly conserved region to which primers can be designed [13] and used to determine conspecific or heterospecific genetic relationships. The analysis of the mtDNA marker cytochrome *c* oxidase gene subunit 1 (*cox*1), is highly discriminatory and has been exploited to confirm monophyletic species across taxa [14]. Due to its maternal inheritance and the ability to accumulate genetic signature, mtDNA is therefore a helpful marker in tracing the genetic patterns and lineages of the *palpalis* group of tsetse flies in Congo Brazzaville. According to Avise [15], analysis of intraspecific mtDNA variation can reveal information about the interconnectivity of populations and past demographic events such as population expansions.

In this study, we investigated the genetic diversity and phylogeography of *palpalis* group flies from three populations (including nine subpopulations) in Congo Brazzaville by sequencing the *cox*1 gene. This gene is used in DNA barcoding as it has a high rate of nucleotide substitution that helps to discriminate cryptic species, reveal novel taxa and resolve relationships between genera [16]. This gene has previously been used to investigate phylogeographic patterns [15] and histories in insects [15, 17], including tsetse flies [14, 18, 19]. For better taxonomic approach and characterization of the *G. fuscipes* (*s.l*.) in Congo Brazzaville, we compared mtDNA *cox*1 sequences generated in this study with sequences of *G. fuscipes* originating from Uganda deposited in the GenBank database [15, 18, 20].

Our overall aim was to validate and better understand the taxonomy and distribution of *G. fuscipes* (*s.l.*) and *G. p. palpalis* in the country as compared to past records based on morphological characterization reported in the literature. Additionally, we aimed to gain insight into the genetic diversity and breeding structure of *G. fuscipes* (*s.l.*) populations for the purpose of designing effective area-wide control strategies at the national level.

## Methods

### Study design and location

**Fig. 1:**
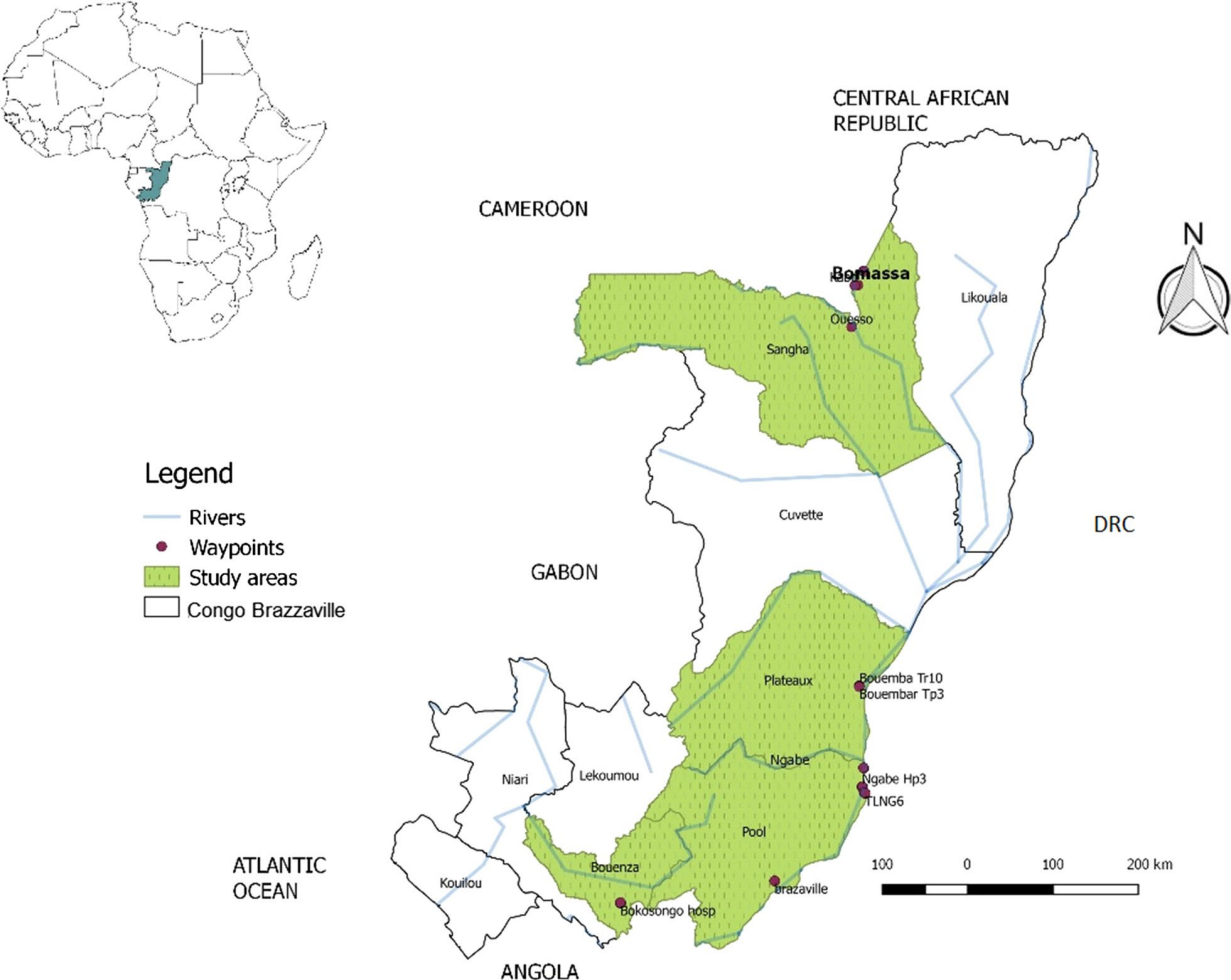
Map of Congo Brazzaville and its neighboring countries, showing tsetse collection sites represented by red dots corresponding to waypoints and Brazzaville the capital. The regions of study are indicated in green: Sangha, plateau and pool region for *G. fuscipes* (*s.l.*) and Bouenza for *G. p. palpalis*

Field sampling of tsetse flies was performed between June and August 2017. Samples of *G. fuscipes* (*s.l.*) were collected from three main regions in Congo Brazzaville with high tsetse density, namely, the Sangha region in the north at the Cameroonian border in Kadei River in BMSA village (2.2039N, 16.1855E), the Plateau region in the HAT focus of BEMB (2.1615S, 16.1363E) and TLG (3.2869S, 16.1968E) in the Ngabe corridor (Fig. 1). Samples of *G. p. palpalis* were obtained from Bokosongo (BKS) located in the Bouenza region of southern Congo. These tsetse infested regions can be grouped into two categories based on the nature of habitat occupied by the flies, i.e. River-Savannah (BKS, TLG and BEMB) and River-Forest (BMSA) (Fig. 1). The sampling area was previously described [11].

### Tsetse fly sample collection

Two hundred and sixty-three *G. fuscipes* (*s.l.*) and 65 *G. p. palpalis* tsetse flies were collected. The flies were caught from along the River Congo and its tributaries. Cross-sectional sampling was used to ensure wide coverage of tsetse infested areas. Four blocks were selected and purposively sampled based on areas reported to be infested with *G. fuscipes* (*s.l.*) and *G. p. palpalis* as per published information on tsetse occurrence and distribution [11, 21, 22] and as communicated by word of mouth (A. Itoua, personal communication). Trapping sites were identified within each block. In the context of this study, the four blocks were BMSA, BEMB, TLG in Kimingui in Ngabe area for *G. fuscipes* (*s.l.*) and Bokosongo (BKS) for *G. p. palpalis.* Ten biconical traps were deployed in each block for seven consecutive days. *Glossina* species were identified morphologically using species identification keys [23]. Fly apparent densities were calculated as number of flies per trap per day (F/T/D), calculated by dividing the total number of tsetse flies captured (ΣF) by the product of the number of functioning traps used to catch them (T) and the number of days for which the traps were operational (D): FTD = ΣF/T × D.

The trapping sites were selected at intervals of at least 300 m and were georeferenced using a GPS handset (Garmin GPS 12 100014600 2.6-inch portable GPS navigator; Garmin, Kansas USA) and included in Google Earth. Non-baited biconical traps [24] were used and catches were collected after every 24 h and flies were counted, aged, sexed and identified morphologically. Sampling was repeated until sufficient tsetse samples were obtained. At least 30 samples (flies) were obtained per collection site. At each sampling block, samples were collected from 3 sampling sites, yielding a total of 96 samples per block and 288 samples for the three blocks for *G. fuscipes* (*s.l.*) before sequence analyses. In the context of this study, sampling sites were considered as putative subpopulations. Thus, there were nine (3 blocks × 3 sites/block = 9) subpopulations of *G. fuscipes* (*s.l*.). For *G. p. palpalis*, we collected 65 samples from two sites in Bokosongo. *G. p. palpalis* samples were only used for determining phylogenetic relationship between *G. fuscipes* (*s.l*.) and *G. p. palpalis.* Therefore, for phylogenetic studies and genetic diversity and differentiation analyses, a total of 328 flies (263 *G. fuscipes* (*s.l*.) and 65 *G. p. palpalis*) were successfully amplified and considered.

Upon obtaining an import permit from the Department of Veterinary Services in Kenya, samples were transported from Congo Brazzaville to the Biotechnology Research Institute of the Kenya Livestock and Agricultural Research Organization (BioRI-KALRO) for further analysis. Samples were preserved in 95% ethanol in 1.5 ml Eppendorf tubes until DNA extraction, amplification and subsequent analyses. Species identification based on morphological characterization was confirmed in the laboratory at BioRI-KALRO using microscopy and based on identification keys for adult *Glossina* species [23]. Samples obtained from a well-characterized *G. f. fuscipes* colony at the International Centre of Insect Physiology and Ecology (ICIPE), Nairobi, Kenya, were used as reference for *G. fuscipes.* Similarly, *G. p. palpalis* samples from the Nigerian Institute of Trypanosomiasis Research (NITR), Kaduna, Nigeria, and served as reference for *G. p. palpalis* from the Central and West African clone and were kindly provided through Professor Soerge Kelm from the University of Bremen, Germany. Unfortunately, even after contacting several institutions, we were unable to obtain characterized reference samples of other *G. fuscipes* taxa (*G. f. quanzensis* or *G. f. martini*).

### DNA extraction, amplification and sequencing

Genomic DNA was extracted from individual tsetse flies by using a DNeasy extraction kit (Qiagen, Maryland, USA) following the manufacturer’s instructions. Briefly, two to three tsetse legs were removed from individual male or female flies that had been preserved in 95% ethanol and air-dried for 10 min. Air-dried legs were dipped into liquid nitrogen and ground using a micro-pestle. The resulting ground tissue was then lysed, precipitated, re-suspended and stored for further DNA extraction and purification as described in the manufacturer’s instructions. The quality and quantity of DNA was checked by using a NanoDrop spectrophotometer to measure the absorbance at 260/280 ratio range.

PCR analysis of mitochondrial DNA for the identification of *G. fuscipes* (*s.l.*) and *G. p. palpalis* using the COI/COII primers was carried out as previously described [13, 18, 25]. From each *G. fuscipes* (*s.l.*) fly, the *cox*1 gene was amplified using 10 µM each of COIF1 (5′-CCT CAA CAC TTT TTA GGT TTA G-3′) and COIIR1 (5′-GGT TCT CTA ATT TCA TCA AGT A-3′) with a final concentration of 0.03 mM each, targeting a 570 bp region. Distilled water and PCR 2× Master Mix AccuPower® (Bioneer, Inc. Amelda, CA, USA) containing Taq DNA polymerase solution were added to a total volume of 30 µl including 5 µl of DNA template added to individual tubes in a 96-well plate. The plates were vortexed shortly for 2 min to ensure total mixing. The amplification was performed using a 2720 Thermal Cycler (Applied Biosystems, Singapore 739256, Singapore) located at BioRI-KALRO, Muguga, Kenya. The cycling conditions were: an initial denaturation at 95 °C for 5 min followed by 37 cycles of 95 °C denaturation for 30 s, 54 °C annealing for 30 s, extension at 72 °C for 45 s. This was followed by a final extension step at 72 °C for 20 min and 4 °C hold.

*Glossina p. palpalis* DNA was amplified at 95 °C for 5 min followed by 35 cycles of 93 °C denaturation for 1 min, 55 °C annealing for 1 min, and extension at 72 °C for 2 min and final extension step at 72 °C for 7 min and 4 °C hold. The primers set used were: 10µM CI-J2195 (5′- TTG ATT TTT TGG TCA TCC AGA AGT-3′) and 10 µM CULR (5′-TGA AGC TTA AAT TCA TTG CAC TAA TC-3′) [26, 27] (primers had a final concentration of 0.05 µM with an expected amplicon size of 850 bp) [28]. Three microliters of PCR products were electrophoresed on a 1.5% agarose gel (0.5× TAE) stained with 1.5 µl Gel Red (10 mg/ml final concentration) and labelled with GelPilot®, 100 bp plus ladder (Cat. No. 239045; Qiagen, Hilden, Germany). Electrophoresis was performed at a constant power supply of 100V for 60 min. The gels were visualized on a UV trans-illuminator and images taken using an UVITEC Cambridge gel system (Uvitec, Brumath, France).

PCR products with tracking dye were then directly submitted for sequencing since Bioneer Taq PCR master mix contains Taq DNA polymerase, with application for direct gene sequencing. One hundred microliters of the PCR product was sent for sequencing to Macrogen Corporation (Microgen Inc, Maryland, and USA).

### Sequence analysis

Raw sequences were processed using the Qiagen CLC Main WorkBench version 7.7.3 software. Both forward and reverse sequences were trimmed, and a consensus obtained before being assembled. Conflicts were solved manually and contigs obtained. All consensus sequences and the output files were then exported as a FASTA format, followed by multiple sequence alignment (MSA) under “Alignment” and “Tree” on “Create Alignment” (CLC) and the implementation of a single file on MEGA7 using default settings. The BLASTn algorithm [20] in the NCBI database was used to blast sequences. The BLASTn return showed homology of fragments with the *cox*1 gene sequences from the GenBank database. The analysis of sequences was performed using DnaSP v5.10.01 [29] for detection of polymorphisms among populations.

### Phylogenetic analysis

*Cox*1 sequences of both *G. fuscipes* (*s.l.*) and *G. p. palpalis* were aligned using MUSCLE [30] and the phylogenetic trees were built. The phylogenic tree of *Glossina* sequences was constructed in MEGA7 [30] and confirmed in MEGA X [31] using Maximum Likelihood (ML) and distance-based trees, using a bootstrap method for 1000 replications to test the best *cox*1 trees and assess node support [32] using the Kimura 2-parameter. Complete deletions were considered as gaps or missing data. The trees were inferred using the ML heuristic method, considering Nearest-Neighbor Interchange (NNI). The initial tree was made automatically by default (NJ/BioNJ) with no branch swap filter. The composition of nucleotides for every sequence was evaluated using MEGA7 [30]. The ML method was used to generate a haplotype genetic tree for *G. fuscipes* (*s.l.*) and also the tsetse tree based on individual sequences. Haplotype sequence analysis for *G. p. palpalis* was not conducted since only one population of *G. p. palpalis* (BKS) was sampled (*vs* three populations of *G. fuscipes* (*s.l.*)).

### Genetic diversity, differentiation and population structure

An input file of *G. fuscipes* (*s.l.*) *cox*1 sequences was generated from CLC Main Workbench version 7.7.3 (Qiagen) and processed in MEGA7 [30]. Thereafter we used DnaSP v6.12.01 [29] to estimate genetic diversity indices and to generate haplotype data file by creating: (i) a haplotype list for Arlequin; and (ii) Roehl data file for Network software. DnaSP was also used to estimate levels of polymorphism within populations. *Cox*1 genetic heterogeneity among populations was quantified by assessing the total number of haplotypes (H), haplotype diversity (Hd), nucleotide diversity (π), and mean nucleotide differences (K) [33]. Hd which is the measure of uniqueness of particular haplotypes in a gene population [34] was evaluated using Nei’s equation 8.4. The average number of nucleotide differences, K [35] (equation A3) was used to measure the uniqueness of a particular haplotype in a given population. Nei’s [36] equation 10.5 was used to calculate nucleotide diversity, Pi (π). Nucleotide diversity is defined as the average number of differences per site between any two sequences chosen randomly from the sampled population. These parameters were all computed using DnaSP [37].

Haplotype distribution and frequencies were obtained and the total number of haplotypes per population was obtained including their sequences and positions. Thereafter, we constructed a median-joining haplotype network [38] using Network 5.0.1.1 [39] to determine the spatial distribution of haplotypes. From each haplotype, pie slices were drawn according to the population of origin and colored depending on the frequencies of specific haplotypes. Genetic differentiation between and among populations was estimated based on fixation index (F_ST_) values as described in [40]. The statistical significance of the total and pairwise fixation indices was estimated by comparing the observed distribution with a null distribution of haplotype frequencies generated by 10,000 permutations. We also performed an analysis of molecular variance (AMOVA) to evaluate the extent to which genetic variation was explained by differences among and within the three *G. fuscipes* (*s.l.*) populations and nine subpopulations. Considering all the nine subpopulations (3 subpopulations per population × 3 populations (BMSA, BEMB and TLG)), groupings were made on the basis of the population of origin. Further population grouping for AMOVA was based on ecological settings (habitats) from where the flies were sampled. For example, we considered that BMSA is located in the rainforest adjacent to a national park whereas BEMB and TLG are located in savannah-river ecological zone.

### Gene flow and demographic history

Rates of gene flow (Nm values) within and among regions/blocks were expressed as the number of female migrants per generation by assuming the island model of population structure [41]. For haploid mitochondrial genome, Nm = (1 – F_ST_)/2F_ST_, with “m” being the female migration rate and “N”, the female effective population size.

We investigated the demographic history of the populations to assess spatial expansions by conducting the neutrality and mismatch distribution tests as implemented in Arlequin and DnaSP. A mismatch distribution is a tabulation of the number of pairwise differences among all DNA sequences in a sample. To test whether the *cox*1 gene sequences conformed to the expectations of neutrality of evolution, we performed Tajima’s D test [42] and Fu’s Fs test of selective neutrality [43], based on the infinite-site model without recombination, and thus appropriate for mtDNA sequences. Tajima’s D test compares the differences between the numbers of segregating sites (S) and the average number of nucleotide differences between two randomly chosen sequences from within the population (K). On the other hand, Fu’s Fs test estimates the probability of observing a random sample with a number of alleles equal to or smaller than the observed value given the observed level of diversity [44]. To test the goodness-of-fit between observed and expected distributions using a model of population expansion we computed the raggedness index (*r*) and the sum of squared deviations (SSD). The nucleotide sequences of 46 haplotypes for *G. fuscipes* (*s.l.*) and representative sequences for *G. p. palpalis* from this study were deposited in the GenBank database under the accession numbers MN586284-MN586591 and MN750696-MN750714, respectively.

## Results

### Tsetse apparent densities

The entomological survey data showed that the apparent density, the number of *G. fuscipes* (*s.l.*) flies per trap per day (FDT) was 16.54 in BMSA, 10.33 in BEMB, and 2.42 in TLG. FTD was 7.86 in BKS for *G. p. palpalis* (Additional file 1: Table S1).

### Morphological characteristics of sampled flies

Based on shape and size of male genitalia (internal claspers) and on size and shape of female dorsal plates [23], the species of tsetse flies caught were identified as *G. f. fuscipes* (in BMSA in Sangha region and TLG in Ngabe corridor) and *G. p. palpalis* (in Bokosongo in south Congo). These two species are closely related and have identical morphological and morphometric features (size and color). The discriminatory criteria used to tell apart the different taxa in the *palpalis* group are based on male genitalia (terminalia) characters, particularly pertaining to the superior and inferior claspers. These criteria are described in [23] and provided in Additional file 2: Text S1. For finer resolution of the identification of the *palpalis* group taxa sampled in this study, we undertook molecular characterization by amplifying and sequencing the mitochondrial *cox*1 gene.

### Molecular characterization of *G. fuscipes* (*s.l.*) and *G. p. palpalis*

PCR amplification of the *cox*1 gene on the morphologically identified *G. fuscipes* (*s.l*.) from BMSA, BEMB and TLG, all northern Congo locations, yielded the expected PCR product (570 bp) as previously described [25]. Similarly, amplification of presumptive *G. p. palpalis* from Bokosongo in southern Congo yielded the expected product (850 bp) based on the primers designed by [26]. Sequencing of *G. fuscipes* (*s.l.*) and *G. p. palpalis cox*1 gene also yielded sequences of 570 bp (Additional file 3: Figure S1a) and 850 bp (Additional file 3: Figure S1b), respectively, after trimming the sequences. These results confirm the presence of both *G. fuscipes* (*s.l*.) and *G. p. palpalis* in Congo Brazzaville.

### Phylogeny, taxonomy and sequence analysis

We examined 263 *G. fuscipes* (*s.l.*) and 64 *G. p. palpalis* sequences after a quality check on CLC Main Workbench version 7.7.3 (Qiagen) as implemented in MEGA 7.0.26 [30]. Sequence analysis showed that *G. fuscipes* (*s.l.*) sequences covered a length of up to 570 bp in BMSA, Bouemba and TLG against 850 bp for *G. p. palpalis* flies from Bokosongo. Comparison of the newly generated sequences using the BLASTn algorithm [20] in the NCBI database with *cox*1 sequences for *Glossina* spp. revealed a high similarity with *G. fuscipes*; BLASTn analysis showed high percentage identities (98.60%, 98.95% and 100% for TLG, BMSA and BEMB, respectively) with specific *G. fuscipes* sequences in the database (all generated from *G. f. fuscipes*) [12, 45]. These percentage identities were recorded with a high total coverage of 97–98%, a total score ranging from 1009–1053 and E-values of zero (0.0), thus demonstrating the authenticity of the data. Altogether, these results demonstrate high levels of similarity between existing database sequences of *G. fuscipes* (originating from *G. f. fuscipes*) and sequences generated from morphologically identified *G. fuscipes* (*s.l.*) samples obtained from TLG, BMSA and BEMB in Congo Brazzaville.

**Fig. 2:**
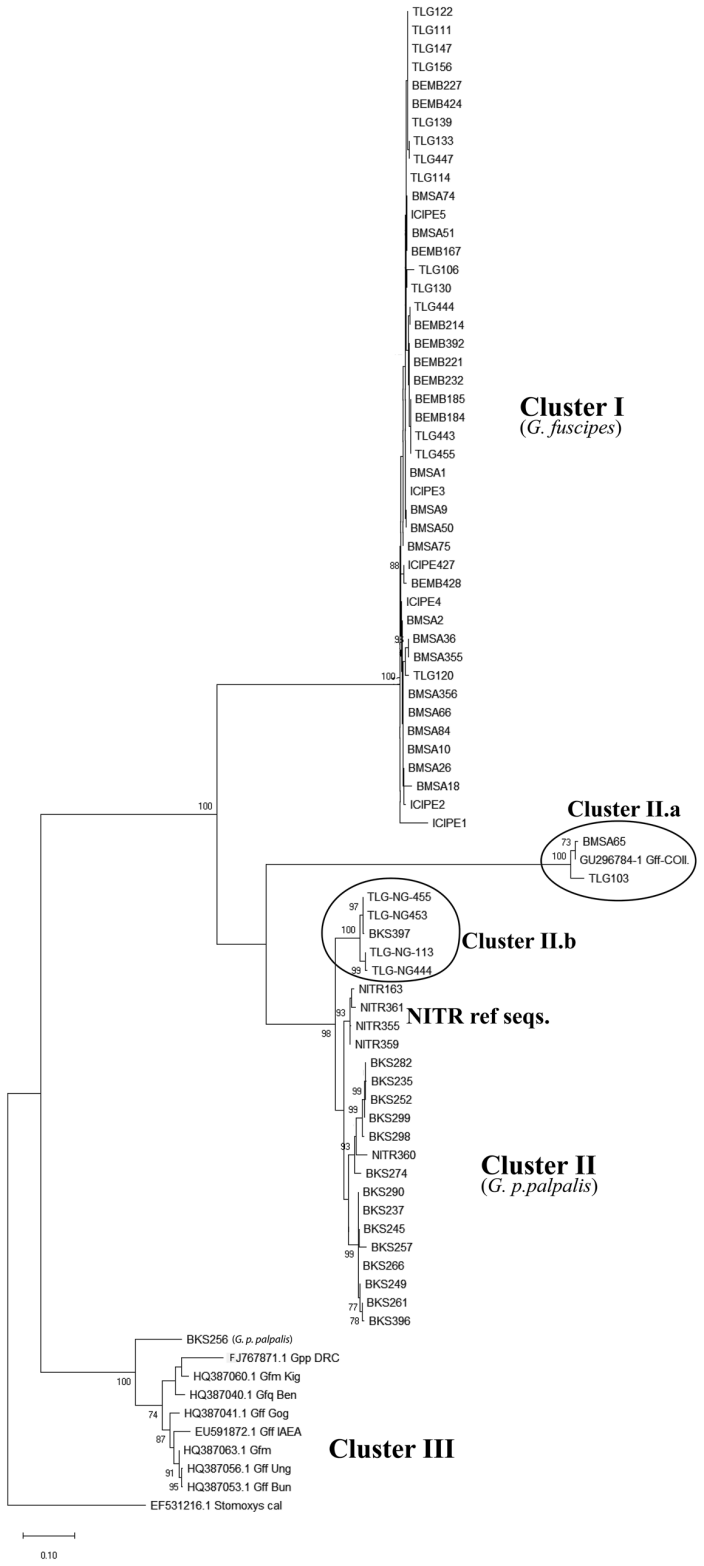
The evolutionary history of *Glossina* mitochondrial sequences showing three main clusters (I, II and III) and a mini-cluster representing particular and atypical *Glossina* sequences (BMSA65 and TLG103). The tree is drawn to scale, with branch lengths measured in the number of substitutions per site. This analysis involved 82 nucleotide representative sequences of *G. fuscipes* (*s.l.*) and *G. p. palpalis*. Evolutionary analyses were conducted in MEGA X [31]. An outgroup sequence of *Stomoxys calcitrans cox*1 gene was included in the analyses. Fig. 2. Selected sequence GenBank accession numbers: MN586290, MN586294, MN586342, MN586345, MN586346, MN586353, MN586355, MN586359, MN586360, MN586393, MN586396, MN586428-MN586433, MN586435-MN586438, MN586462, MN586489-MN586491, MN586514, MN586518, MN586575, MN586579-MN586583, MN586585-MN586588, MN586590, MN586591

We observed two main clusters of *Glossina* species in this study, one representing *G. fuscipes* (*s.l.*) (Fig. 2) and the other representing *G. p. palpalis.* Whereas *G. fuscipes* (*s.l.*), represented by TLG103 and BMSA65, had a perfect match of 99.8% with *G. f. fuscipes* (GenBank: GU296784.1) from the neighboring DRC, a *G. p. palpalis* sample from Bokosongo (BKS256) had a common ancestry with a sequence of *G. p. palpalis*. Reference *G. f. fuscipes* samples from ICIPE (Kenya) clustered with the *G. fuscipes* (*s.l.*) samples collected from field sites in Congo Brazzaville. Similarly, *G. p. palpalis* reference samples from NITR (Nigeria) clustered with *G. p. palpalis* samples from Bokosongo, thus confirming the existence of both *G. fuscipes* (*s.l*.) and *G. p. palpalis* and their distinct habitats in Congo Brazzaville. Cluster I (Fig. 2) shows a mixture of individuals from BEMB, TLG and BMSA clustering with reference *G. f. fuscipes* samples from ICIPE. Interestingly, some putative *G. p. palpalis* sequences clustered in TLG, a known locality of *G. fuscipes.* Cluster IIa shows some individuals from BMSA and TLG clustering with documented *G. fuscipes* (*s.l*.) from GenBank. Cluster II (Fig. 2) represents *G. p. palpalis* from Bokosongo (BKS). Strangely, Cluster IIa also includes a published *G. fuscipes* sequence (GenBank: GU296784.1) [18]. Surprisingly, sample BKS256 from BKS, a *G. p. palpalis* region, shares common ancestry with published sequences for *G. f. quanzensis*, *G. f. martini* and *G. p. palpalis* from neighboring DRC (GenBank: FJ767871.1) Fig. 2 (Cluster III). Taken together, these results show that the phylogeny/taxonomy of the *palpalis* group of tsetse flies in Congo Brazzaville is much more complex and requires further studies to be fully resolved.

### Genetic diversity and haplotype distribution

Overall, we observed high haplotype diversity (Hd = 0.884) and moderate nucleotide diversity ( = 0.012) at 204 segregating sites in the three sub-lineages. The total number of haplotypes observed was 46. Diversity indices varied among populations (Table 1**)**. The number of haplotypes per population (*h*) ranged from 9 in BEMB to 25 in BMSA. In TLG *h* was 21. Haplotype diversity varied from 0.582 in BEMB to 0.860 in BMSA with TLG recording Hd of 0.812. Nucleotide diversity () was lowest in BEMB ( = 0.002) and marginally higher in TLG ( = 0.018) than in BMSA ( = 0.016). Out of the 263 sequences examined, the number of segregating sites (S) was 165, 9 and 186 in BMSA, BEMB and TLG, respectively. The average number of haplotype differences (K) for the three populations was, respectively, 7.09, 0.99 and 6.82. Overall, these results show that diversities were highest in BMSA and lowest in BEMB.

**Table 1:** Genetic diversity indices, test of selective neutrality, goodness-of-fit-test

Population	No. of sequences (No. of haplotypes)	S	Pi	Hd	Neutrality test		Goodness-of-fit	
					Tajima’s D	Fu’s Fs	SSD	Hrag
Bouemba	88 (9)	0.9882	0.0022	0.5815	− 1.13378 (*P* = 0.13)	− 3.22268 (*P* = 0.086)	0.00224 (*P* = 0.568)	0.05537 (*P* = 0.706)
Talangai	87 (21)	6.8209	0.0152	0.8062	− **2.76701 (*P*< 0.0001)**	− 1.05260 (*P* = 0.420**)**	0.01219 (*P* = 0.778)	0.02424 (*P* = 0.866)
Bomassa	88 (25)	7.0867	0.0158	0.8602	− **2.64837 (*P <*0.0001)**	− 2.86023 (*P* = 0.2180)	0.02735 (*P* = 0.178)	0.06618 (*P* = 0.115)

*Note*: Tajima’s D, significance of *P* < 0.01 is highlighted in bold. Number of haplotypes and Tajima’s D, Fu’s Fs, SSD and Hrag *P*-values are in parentheses

*Abbreviations*: S, number of segregating sites; Pi, nucleotide diversity; Hd, haplotype diversity; SSD, sum of squared deviation between the observed and expected distribution of pairwise differences; Hrag, Harpending’s raggedness index (non-significant, data have relatively good fit to a model of population expansion) and a ragged distribution suggests that the lineage was widespread

**Fig. 3:**
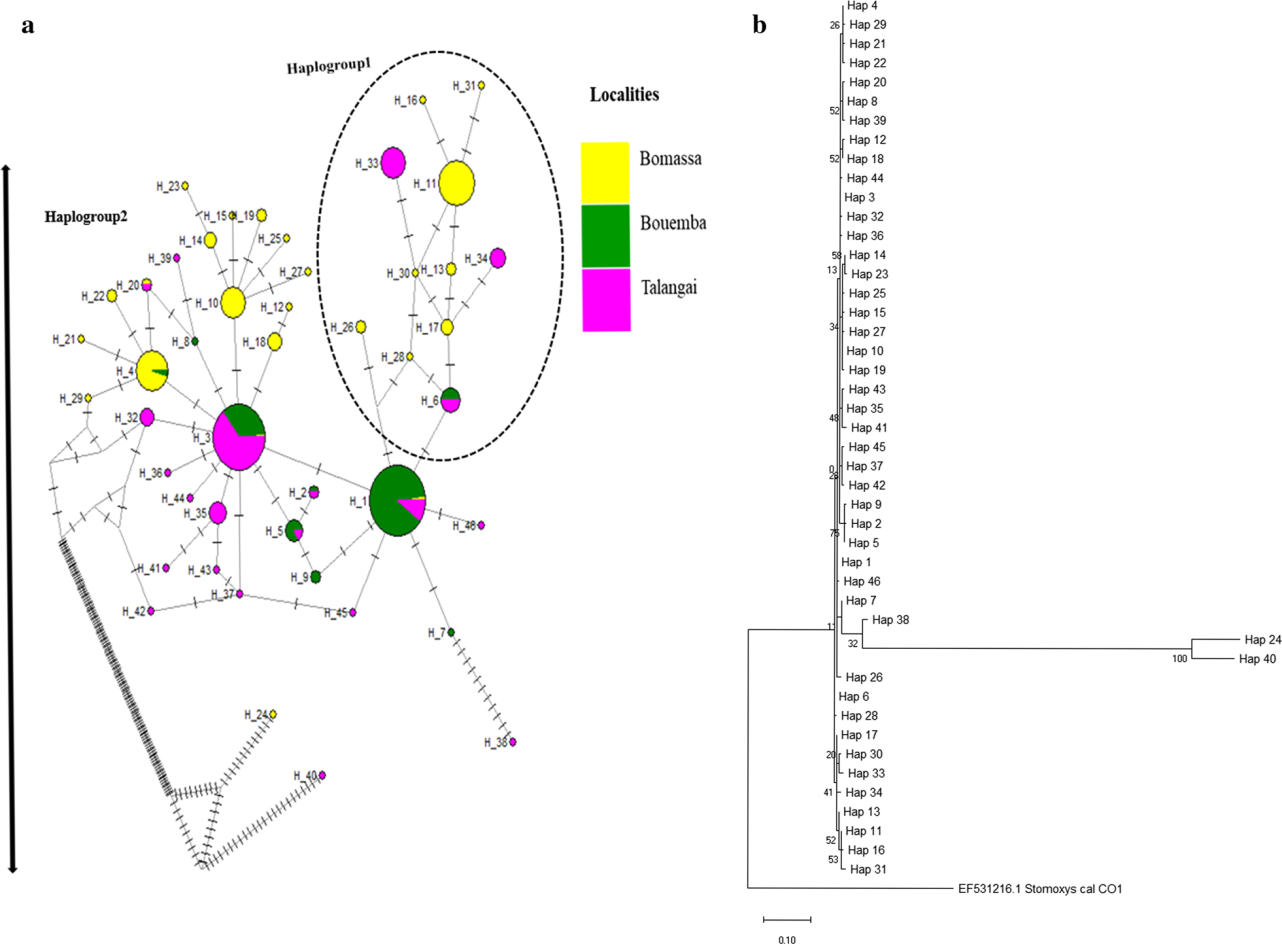
Median joining network (MJN) and evolutionary history of 46 mitochondrial *cox*1 haplotypes between the three *G. fuscipes* populations (BMSA, BEMB and TLG). **a** Haplotype variations in 3 populations of *G. fuscipes* (*s.l.*) infested areas (BMSA, BEMB and TLG). Each circle (node) represents haplotype and sizes are proportional to their frequencies of sampled individual sequences type (1–46). **b** The evolutionary history of mitochondrial *cox*1 haplotypes between the three *G. fuscipes* (*s.l.*) populations (BMSA, BEMB and TLG) based on haplotype distribution. The analysis involved 46 nucleotide sequences and a total of 216 positions in the final dataset

Figure 3a shows the genetic divergence and haplotype network among the three populations as produced by the median joining network (MJN), which is indeed complementary to Fig. 3b. From the 263 mitochondrial *cox*1 sequences analyzed, we obtained 46 haplotypes distributed among the three populations (Fig. 3b). A significant proportion (84.78%) of the haplotypes in the three localities were private (found in only one population). Only 15.22% of the haplotypes were shared among populations. BMSA had the highest proportion of private haplotypes (47.83%) followed by TLG (30.43%) and BEMB with only 6.52%. Of the shared haplotypes, 6.52% were shared between BEMB and TLG, 4.35% between BMSA, BEMB and TLG and 2.17% between BMSA and BEMB. The network shows two main haplogroups (Fig. 3b). The first is constituted predominantly of private haplotypes from BMSA and TLG, with its base made of shared ancestral haplotypes (H1 and H6) shared between the two populations. The second main haplogroup is made up of both shared and private haplotypes from all locations, characterized by the star-like network radiating from BMSA and having emerged from two private haplotypes (H4 and H10), indicating the expansion of *G. fuscipes* (*s.l.*) population in BMSA. On the other hand, BEMB is constituted with trapped subnet with no free nodes of private haplotypes. All sub-networks are continuous and connected, indicating that BEMB is mainly populated by mixed haplotypes as well as the remaining private haplotypes from all localities. Colors for each pie slices represent locations of sample origin of collections and their size. Private haplotypes with a unique color and shared haplotypes with a mixed color (haplotype sequences) and dominant haplotypes with higher haplotype frequency, mixed colors (shared) and single color (private). The parallel lines represent mutations which have occurred in each haplotype of the sampled sequences at nucleotide positions (Fig. 3.)

### Genetic differentiation and structure

Pairwise F_ST_ values among the three analyzed populations are presented in Additional file 4: Table S2. BMSA and BEMB were the most genetically differentiated populations (F_ST_ = 0.152, *P* = 0.000). Levels of genetic differentiation were low between BMSA and TLG and between BEMB and TLG with F_ST_ values of 0.048 and 0.072, respectively. These indices indicate low to moderate genetic differentiation among the *G. fuscipes* (*s.l*.) populations sampled in Congo Brazzaville. These results thus indicate restricted exchange of genetic material between BEMB and BMSA and almost unlimited gene flow between TLG and BMSA or TLG and BEMB. Values of genetic differentiation were estimated by using Wright’s fixation index (F_ST_) [45].

Pairwise genetic distances among subpopulations within population groups were low and insignificant. This was true for subpopulations within BEMB (group 1), TLG (group 2) and BMSA (group 3) (Additional file 5: Table S3), indicating absence of genetic subdivision among subpopulations within populations.

Analysis of molecular variance (AMOVA) indicated that ~13% of the genetic variation was attributed to differences among the three populations studied (BMSA, BEMB and TLG). Less than 1% of the genetic variance was due to differences among subpopulations within populations, whereas most of the genetic variance (~86%) was explained by differences among individuals within subpopulations (Table 2). The corresponding F-statistics indicate moderate and significant genetic differentiation among the three populations (F_CT (2, 262)_ = 0.14, *P* = 0.003) and unlimited gene flow among subpopulations within populations (F_SC (6, 262)_ ~0.010, *P* = 0.046).

**Table 2:** Molecular analysis of variance (AMOVA) of the nine subpopulations of BMSA, BEMB and TLG based on distance method structure

Source of variation	*df*	Sum of squares	Variance components	Percent variation	Fixation index (*P*-value)
Among groups	2	171.864	0.89603 (Va)	13.4	F_CT_ = 0.14 (*P* = 0.003)
Among subpopulations within groups	6	44.265	0.05553 (Vb)	0.83	F_SC_ = 0.01 (*P* = 0.046)
Among individuals within populations	254	1461.985	5.75585 (Vc)	85.8	F_ST_ = 0.14 (*P* < 0.0001)
Total	262	1678.114	6.70741	100	

*Abbreviations*: *df*, degrees of freedom; Va, variance of a; Vb, variance of b; Vc, variance of c; F_CT_, tested under random permutations of whole populations across regions; F_SC_, tested under random permutation of individuals across populations but within the same region; F_ST_, tested under random permutation of individuals across populations regardless of either their original populations or regions

When subpopulations were grouped according to ecological conditions, the variance due to ecological differences was ~8% whereas the differences attributed to subpopulations within ecological groups (Additional file 6: Table S4) was only 2.35%. Most (89.7%) of the variance lay among individuals within subpopulations.

### Demographic history

Neutrality test based on Fu’s Fs and Tajima’s D tests (Table 1) conducted to give insights into demographic dynamics show negative values in all populations. The mean D value across the three populations was negative (-2.18305, *P* < 0.05). Additionally, D values for TLG and BMSA were negative and statistically significant (*P* < 0.01), suggesting recent population expansions. In BEMB, however, Tajima’s D was negative but not significant (*P* > 0.1). Similarly, Fu’s Fs values in each of the three populations and overall, were negative but not significant.

The sums of squared deviations (SSD) and Harpending’s raggedness index I-values and their associated *P*-values (Table 1) suggested goodness-of-fit between the observed and the expected distributions, thus lending further evidence to population expansion. The mismatch distribution results are shown in Additional file 7: Figure S2a-c. Additional file 8: Table S5 shows mitochondrial *cox*1 gene haplotype IDs, frequencies and order positions for *G. fuscipes* (*s.l.*) from Congo Brazzaville and GenBank accession numbers for sequences, sample codes and sample metadata are provided in provided in Additional file 9: Table S6 and related sequences GenBank accession numbers and sequences, sample codes and ID .b Project/Code, sequences processed ID and Sequence length are shown in Additional File 10: Table S7a-b.

## Discussion

### Tsetse taxonomic status

The genetic taxonomic status of *palpalis* group tsetse flies in Congo Brazzaville, particularly in the areas of Bomassa (BMSA) in the north, Bouemba (BEMB) and Talangai (TLG) in Plateau Batéké and in Bokosongo (BKS) in south Congo, has been largely unknown to date. Previous morphological characterization had classified existing *Glossina* taxa in the country into *G. f. quanzensis*, *G. f. martini* and *G. f. fuscipes*, and *G. p. palpalis* [7, 22]. Morphological characterization [46] in the present study demonstrated the presence of putative *G. fuscipes* (*s.l.*) in BMSA, BEMB and TLG and *G. p. palpalis* in the only site of BKS (in the south) based on features of the male genitalia using identification keys (Additional file 2: Text S1) for *Glossina* spp. [23]. It should be noted however, that classification based on morphological characters (e.g. male genitalia) is complex and has previously led to a misclassification of certain *Glossina* taxa [22]. These results should therefore be interpreted with caution.

To the best of our knowledge, this is the first report on phylogenetic relationships of *palpalis* group flies in Congo Brazzaville based on *cox*1 gene sequences. The data we have generated confirm the presence of both *G. fuscipes* (*s.l.*) and *G. p. palpalis* [46] as demonstrated by successful amplification of mtDNA fragments of 570 bp (Additional file 3: Figure S1a) and 850 bp (Additional file 3: Figure S1b), respectively, using primers previously developed and validated for *G. fuscipes* (*s.l.*) [25] and *G. p. palpalis* [47]. Our results are consistent with earlier findings from morphological studies that reported the presence of *G. fuscipes* (*s.l.*) and *G. p. palpalis* in Congo Brazzaville [22].

The finding of *G. fuscipes* (*s.l.*) in BMSA along the Kadei River [4, 48, 49], in TLG in the Ngabe corridor, and in BEMB in plateau Batéké, confirms their presence in the Congo Basin habitats*. Glossina fuscipes* is distributed in all hydrographical systems/networks in the Congo region and is hypothesized to have adapted to ecological conditions in south-east Cameroon, region at the Congo-Cameroon border [50]. The distribution of *G. p. palpalis* identified in BKS apparently stretches from Djoué River to the southern part of Congo [7, 22]. It is likely that this subspecies was the one previously morphologically mischaracterized and classified as being a distinct species from *G. f. fuscipes* found in the Ngabe Corridor (BEMB and TLG) [22]. The upper Pool region is reported to be colonized by *G. f. quanzensis* [51, 52] in the Ngabe corridor.

The phylogenetic analysis showed two main clusters separating *G. fuscipes* (*s.l.*) and *G. p. palpalis*, with several sub-clusters within the main groups and a mixture of individuals from all localities (Fig. 2). This is indicative of unrestricted dispersal of *palpalis* group tsetse flies within the country. As would be expected, the reference samples of *G. f. fuscipes* and *G. p. palpalis* obtained from ICIPE (Kenya) and NITR (Nigeria) clustered, respectively, with *G. fuscipes* (*s.l.*) and *G. p. palpalis* collected from areas of their expected natural distribution in Congo Brazzaville. Based on phylogenetic and BLASTn analysis, our results showed 100% homology of sequences in BEMB (sample BEMB402) with *G. fuscipes* haplotypes (GenBank: GU296784.1) found in Uganda [18]. Although deposited in GenBank as *G. fuscipes*, this sequence from Uganda originated from samples morphologically classified as *G. f. fuscipes.* In BMSA and TLG we observed 98.95% and 98.6% identities, respectively with GenBank sequences of *G. fuscipes.* These results indicate that whereas BEMB seems to be inhabited by *G. f. fuscipes* (100% similarity to East African *G. f. fuscipes* from Uganda), BMSA and TLG could be populated with a *G. fuscipes* complex composed of *G. f. quanzensis*, *G. f. martini* and *G. f. fuscipes* as previously reported in the Ngabe corridor and in the northern part of the country [7, 11, 22, 51, 52]. The sequence of sample BKS256 (presumed to be *G. p. palpalis*) for instance, clustered with reference *G*. *fuscipes* sequences from GenBank (Cluster III, Fig. 2), all putative *G*. *fuscipes* sampled in BMSA, BEMB and TLG clustered together in Cluster I. As would be expected, a GenBank sequence identified as *G. p. palpalis* from DRC (GenBank: FJ767871.1) also clustered with BKS256 and curiously with other *G. fuscipes* (*s.l.*) sequences from the database as well. These findings send mixed signals. First, it is possible that both BKS256 and EU591840-2 are *G. p. palpalis* and not *G. fuscipes* (*s.l*.). Secondly, it can be hypothesized that BKS256 is indeed *G. p. palpalis* that could have recently dispersed into a predominantly *G. fuscipes* (*s.l*.) habitat, consistent with previous claims of an overlap in the distribution of the two taxa [11]. It is apparent that the *G. fuscipes* (*s.l*.) obtained from BMSA, BEMB and TLG (making up Cluster I) (Fig. 3) have clearly diverged from *G. p. palpalis* BKS (Cluster II), or perhaps we are dealing with different subspecies of *G. fuscipes* such as *G. f. quanzensis* or *G. f. martini*. These two subspecies are known to share common ancestry [7, 53] and seem to be more closely related with *G. palpalis* as demonstrated by the clustering together with *G. p. palpalis* from DRC with GenBank sequences of *G. quanzensis* and *G. martini*.

Our study has provided prilimirary results that justify that indeed, *G. fuscipes* (*s.l*.) and *G. p. palpalis* are very closely related subspecies but have differences at the molecular level which were not previously established and reported in Congo Brazzaville. We have also demonstrated that *G. fuscipes* (*s.l.*) in BEMB shares high identity (100%) to *G. f. fuscipes* in East Africa, and therefore it is likely that BEMB is colonized by *G. f. fuscipes* whereas BMSA and TLG are colonized by other taxa of *G. fuscipes* (*s.l*.) e.g. *G. f. quanzensis* and *G. f. martini*. However, this will need to be confirmed through further studies in future.

### Haplotype and nucleotide diversities

The present study shows that the sampled *G. fuscipes* (*s.l.*) populations exhibited high haplotype diversity but low nucleotide diversity and moderate nucleotide differences across the three *G. fuscipes* sub-lineages in the Congo. The high haplotype diversity and low nucleotide diversity across all samples is an indication of a rapid demographic expansion [15]. The high haplotype diversity (Table 2) observed in BMSA and the private haplotype richness are indicative of a large effective population size in this area.

The low nucleotide diversity in the three expanding populations is likely due to previous bottlenecks that affected nucleotide diversity. Populations of *G. fuscipes* (*s.l.*) in the Congo seem to be undergoing expansion/repopulations after bottleneck pressures of the past, which could have occurred in BMSA and TLG, but not in BEMB perhaps as result of the long eradication efforts [8, 54, 56] for over five decades in the country [8].

Nucleotide diversity is more likely to be affected by the number of segregating sites than haplotype diversity, and the number of segregating sites is more affected by the size of the current rather than historical populations [42]. This could be the case in TLG and BMSA where the number of segregating sites seems to recover much faster. It would appear that the number of segregating sites (Table 1) in BEMB (S = 0.988) is influenced by the size of the current population more strongly than is the average number of nucleotide differences. On the other hand, the average number of nucleotide differences is affected by the size of the original population more severely than is the number of segregating sites. We hypothesize that the low number of segregating sites (Table 1) and low nucleotide diversity (Pi () = 0.002) observed in BEMB could have resulted from a past severe bottleneck, characterized by low haplotype diversity (Hd = 0.52), with 9 haplotypes among which 2 are private and 7 are shared (Fig. 3a). Such bottlenecks could have been caused by habitat fragmentation, intense control efforts [8, 54], or disappearance of vertebrates that serve as sources of blood meal [55] for the flies. Indeed, large infrastructure or major public works projects in the country such as the complete transformation of the Ngabe village into a township, could have caused habitat fragmentation, leading to a bottleneck [56]. The extremely low number of unique haplotypes (Fig. 3a) and variable sites (Table 1) could be an indicator of extreme demographic declines. Low genetic diversity due to bottlenecks has previously been reported among *morsitans* group tsetse flies in southern Africa [44]. A transient bottleneck was reported in [57] to have resulted from a rinderpest panzootic that occurred in southern Africa in the early 20th century, killing nearly 90% of the mammalian fauna thereby greatly reducing tsetse fly opportunity to obtain blood meals. It would seem that this bottleneck also affected the genetic diversity of the tsetse flies of the *G*. *palpalis* group in central Africa.

### Genetic differentiation and population structure

Pairwise F_ST_ values based on haplotype frequencies and genetic divergence among the nine subpopulations were not significant between BEMB and TLG and between TLG and BMSA subpopulations. Low F_ST_ values observed between these subpopulations indicate high rates of maternal gene flow among them, particularly through the network of rivers such as Kadei, Congo and Sangha Rivers. However, higher and significant F_ST_ values (F_ST_ = 0.152, *P* < 0.05) were observed between BMSA and BEMB. This high genetic differentiation could be attributed to the large geographical distance (400 km) between the two locations and difference in tributaries of the rivers passing through the two regions. AMOVA results indicated considerable subdivision of *G. fuscipes* (*s.l.*) populations in Congo Brazzaville with ~13% and 8% of the genetic variation being attributed to differences among the three populations studied (BMSA, BEMB and TLG) when grouped according to region (north or south) or ecological conditions of habitats from where samples were collected. There was hardly any subdivision among the subpopulations and most of the genetic variance (~86%) was attributed to differences among individuals within subpopulations. The low structuring of *G. fuscipes* (*s.l.*) populations is indicative of ongoing maternal gene flow among the populations regardless of ecological settings.

### Demographic history

The analysis of geographical variation of mitochondrial *cox*1 haplotypes showed a composite structure of the two identified lineages of *G. fuscipes* (*s.l.*) in Congo Brazzaville. A high rate of private haplotypes was observed in BMSA and TLG subpopulations (Fig 3a). Some of the haplotypes are specific to the BMSA region, indicating restricted gene flow from BMSA and a population that is adapted to local conditions [58]. The neutrality tests of evolution yielded negative Tajima’s D values in BEMB, TLG and BMSA, indicating excess of observed rare nucleotide site variants compared to what is expected under neutral model of evolution [59]. These deviations from neutrality are significant only for TLG and BMSA but not in BEMB. A negative Tajimaʼs D value is signature of a recent population expansion in all localities. The Fu’s Fs values, which rely on haplotype distribution were also negative in all populations, a result attributable to excess numbers of rare haplotypes compared to the values expected under neutral evolution. Thus, negative Tajimaʼs D and Fu’s Fs values in all populations (Table 1) revealed signatures of recent and rapid demographic expansion from small effective sizes [59].

The mismatch distribution analysis (Additional file 7: Figure S2a-c) showed past population expansion in TLG and BMSA and the bottlenecks in population size observed in BEMB. Bottlenecks in population size also generate waves, similar to those produced by a sudden expansion [60].

### Geographical distribution of diversity

Ancestral haplotypes were observed more frequently in BEMB than in the other populations (BMSA and TLG, Fig. 3a). BMSA population shared fewer haplotypes with other localities perhaps because the environmental conditions (primarily rainforest) in this area are so specific and therefore not suitable to the lineages found in BEMB and TLG. Climatic conditions in BMSA are characterized by recurrent rainfall throughout the year. The rate of mutations observed for H24 and H40 (Fig. 3b) may be a result of evolutionary forces, such as population growth. The low haplotype richness and diversity in BEMB, associated with predominance of shared haplotypes and low frequency of private haplotypes could be related to the population undergoing or recovering from bottleneck.

The BEMB population could be the oldest lineage among the three populations. The expansion of populations in BMSA and TLG could be attributed to the high numbers of wild and domestic animals in these locations, as opposed to BEMB where there has been intensive human environmental interference and rarity of domestic and wild animals. The intensive human activities especially at the borders of BEMB and TLG in addition to the ongoing countrywide municipal public works programme on road infrastructure and construction of buildings, could have changed tsetse ecology and distribution in the country. Indeed, it has been reported that changes in the environment do affect tsetse density and distribution [61], and could negatively impact demographic and morphological parameters, thus stressing the population and leading to changes in its structure [56].

The shape of the network structure (Fig. 3a), showing a star-like pattern in BMSA, confirms a signal of a recent population expansion, with most of the haplotypes originating from a single parent [62]. The expansion seems to originate from mixed haplotypes (H1 and H3), which could be understood as the genetic backup [61] of the BMSA population. This may suggest that populations at BEMB and BMSA were isolated in the past as they are still today. Haplogroup 1 indicated that a low expansion is also occurring out of TLG and most of the haplotypes that could have been lost during a past bottleneck are being recovered, perhaps due to the high rate of gene flow between TLG and BM SA [62]. H24 and H40 are private haplotypes which evolved from BMSA65 and TLG103 individual isolates (GenBank metadata are shown in Additional file 8: Table S6a-b).

## Conclusions

To the best of our knowledge, this study constitutes the first report on the genetic taxonomic status, diversity, and phylogenetic relationships of *G. fuscipes* (*s.l.*) populations in Talangai (TLG, Ngabe corridor), Bouemba (BEMB) in plateau Batéké up to Bomassa (BMSA), in Sangha region in the northern Congo), based on the mtDNA *cox*1 marker. We have confirmed the presence of *G. fuscipes* at the species level (*G. fuscipes* (*s.l.*)) in northern Congo Brazzaville and the presence of *G. p. palpalis* in southern Congo Brazzaville, particularly in the Bokosongo area. A much more widespread sampling scheme is recommended to establish the full distribution of both *G. fuscipes* (*s.l.*) and *G. p. palpalis* in Congo Brazzaville. The phylogenetic analysis showed that the two taxa of *palpalis* group (*G. fuscipes* (*s.l.*) and *G. p. palpalis*) in Congo Brazzaville are closely related (> 99% similarity) and only have minor differences. All the three populations sampled in this study exhibited high haplotype and low nucleotide diversities, an indication that the populations are recovering from recent bottleneck events. Whereas BEMB and BMSA populations were significantly differentiated genetically due to the large geographical distance between them, subpopulations within BEMB, BMSA and TLG seem to exchange genetic material freely. Thus, any control efforts should consider the population and not the subpopulation as the smallest unit to be targeted for eradication by using area-wide approaches such as the sterile insect technique. The high proportion of private haplotypes in the populations from BMSA and TLG and the high rate of singletons in those from BMSA are indicative of haplotypes that are adapted to the local conditions. The rates observed in the mtDNA network structure showed that *G. fuscipes* (*s.l*.) populations were linked in the past and have undergone recent rapid demographic expansion. The results suggest a population bottleneck in BEMB and TLG that is likely associated with the low genetic diversity observed in the two localities.

## Supplementary information

**Additional file 1: Table S1.** Summary of entomological data and the mean number of flies per trap per day (FTD), showing absolute number of flies, using the equation FTD **=** ΣF/T × D.

**Additional file 2: Text S1.** Morphological characterization key for *Glossina* of the *palpalis* group.

**Additional file 3: Figure S1. a** Amplification of the *cox*1 gene for *G. fuscipes* (*s.l.*) using the COIF1/COIR1 primer set. Expected amplicon size: 570 bp. **b** Amplification of *cox*1 gene for *G. p. palpalis* using the CI-J-2195/CULR primer set. Expected amplicon size: 850 bp.

**Additional file 4: Table S2.** Fixation index (F_ST_) based on haplotype frequencies between the three populations of BMSA and TLG and BEMB.

**Additional file 5: Table S3.** Pairwise F_ST_ values among nine *G. f. fuscipes* subpopulations from three population groups in Congo Brazzaville. Values written in bold represent significant pairwise differentiation (*p* < 0.05). F_ST_*P*-values, matrix of significant F_ST_*P*-values, significance level = 0.05 are highlighted in bold.

**Additional file 6: Table S4.** Hierarchical analysis of molecular variance (AMOVA) design and results based on distance method of genetic structure [40]. Pairwise difference based on the two ecological localities, Plateau Batéké (BEMB-TLG) against (BMSA) rainforest, to test whether the genetic differentiation is influenced by ecology or environmental conditions/factors.

**Additional file 7: Figure S2.** Mismatch distribution of haplotype pairwise nucleotide differences for *G. fuscipes* (*s.l.*) in the three localities, BEMB (**a**), TLG (**b**) and BMSA (**c**), showing observed (red lines) and expected (green lines) frequencies obtained under a model following populations’ size change.

**Additional file 8: Table S5.** Mitochondrial *cox*1 gene haplotype IDs, frequencies and order positions for *G. fuscipes* (*s.l.*) from Congo Brazzaville.

**Additional file 9: Table S6**. *Glossina fuscipes s*amples metadata files including Laboratory datasheet, voucher informations, taxonomy, specimen details and collection data.

**Additional File 10: Table S7. a***Glossina fuscipes* GenBank accession numbers and sequences, sample codes and ID. **b** Project/Code, sequences processed ID and sequence length.

## Data Availability

All data generated and analyzed during this study are included in this article and its additional files. Sequence data are deposited in the GenBank database under the accession numbers MN586284-MN586591 (*G. fuscipes*) and MN750696-MN750714 (*G. p. palpalis*). DNA material and sample carcasses were stored at Kenya Agricultural and Livestock Research Organization-Biotechnology Research Institute.

## Abbreviations

mtDNA: mitochondrial DNA
*cox*1: cytochrome *c* oxidase subunit 1
*cox*2: cytochrome *c* oxidase subunit 2
BMSA: Bomassa
BEMB: Bouemba
TLG: Talangai
BKS: Bokosongo
F_ST_: fixation index
Pi (π): nucleotide diversity
Hd: haplotype diversity
H4: haplotype no. 4
SSD: sum of squared deviations
FTD: mean number of flies per trap per day
BLAST: Basic Local Alignment Search Tool
