# Supplementary material for: Genetic diversity and phylogenetic relationships of tsetse flies of the palpalis group in Congo Brazzaville based on mitochondrial cox1 gene sequences
Source: Parasit Vectors. 2020 May 14;13:253. doi: 10.1186/s13071-020-04120-3 (PMC7227191; doi:10.1186/s13071-020-04120-3)
Supplement: Supplementary file 1 — Additional file 1: Table S1. Summary of entomological data and the mean number of flies per trap per day (FTD), showing absolute number of flies, using the equation FTD = ΣF/T × D. [file 13071_2020_4120_MOESM1_ESM.docx]

**Additional file 1: Table S1**. Summary of entomological data and the Mean number of flies per trap per day (FTD), showing absolute number of flies.

| **Area of collection** | **Collection**  **Days** | **Trap/site** | **Glossina species** | **Sex** | | **Total** | **FTD** |
| --- | --- | --- | --- | --- | --- | --- | --- |
|  |  |  |  | M | F |  |  |
| **Bouemba** | 7 | 10 | *Gf f* | 410 | 313 | 723 | 10.33 |
| **Ngabe/Talangai** | 7 | 10 | *Gff* | 66 | 104 | 170 | 2.42 |
|  |  |  | *Unknown* | 0 | 5 | 5 | 0.07 |
| **Bomassa** | 7 | 10 | *Gff* | 236 | 95 | 1158 | 16.54 |
| **Bokosongo** | 7 | 10 | *Gpp* | 218 | 332 | 550 | 7.86 |
| **Total No. of flies** |  | | | | | **2,606** | 37.22 |
