# Supplementary material for: Genetic diversity and phylogenetic relationships of tsetse flies of the palpalis group in Congo Brazzaville based on mitochondrial cox1 gene sequences
Source: Parasit Vectors. 2020 May 14;13:253. doi: 10.1186/s13071-020-04120-3 (PMC7227191; doi:10.1186/s13071-020-04120-3)
Supplement: Supplementary file 2 — Additional file 2: Text S1. Morphological characterization key for Glossina of the palpalis group. [file 13071_2020_4120_MOESM2_ESM.pdf]

Additional File 2: Text S1

MORPHOLOGICAL CHARACTERIZATION METHOD

Morphological characteristics of Tsetse populations from Congo Brazzaville in the selected areas was performed in the field as described by Itoua *et al.2006* and later confirmed at KALRO-Biotechnology Research Institute in Muguga Kenya following the identification list of key. The method consist of list two questions and by elimination of one of the statements((Leak, Ejigu, & Vreysen, 2008).Briefly, for the *palpalis* group, by the Characters of the Male Terminalia,

Superior claspers of male Glossina of the palpalis Group (Source: Mulligan 1970)

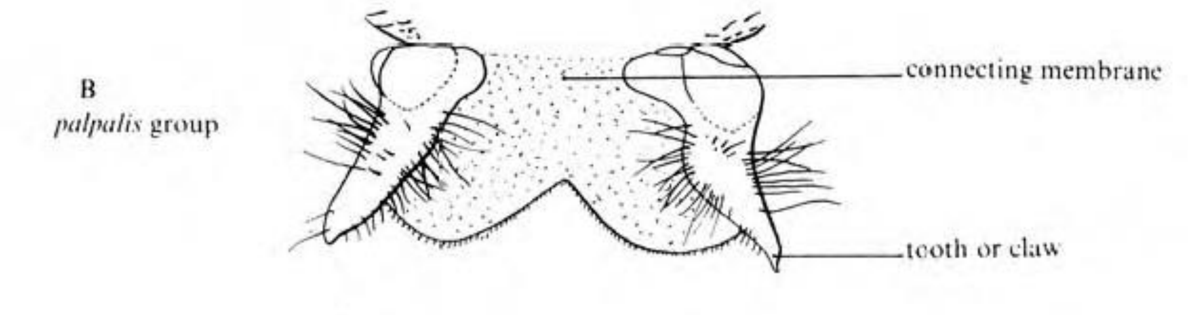

Outer lateral angles of superior claspers rounded and not strongly produced and median processes not prominent and generally projecting only slightly if at all between the inferior claspers (Figure..a,d) and never projecting for more than their length (Figure 1.33b,c).Processes of harpes simple, not bifid **and n**one of the three pairs of processes with bifid members (**Figures d,f**) Harpes none of the processes dilated distally; pairs of processes not all of approximately
the same length (**Figures d, 1.34d**) .Superior claspers joined by a membrane; may terminate in a tooth or claw as in preceding (Figure 1.28b).. Superior claspers terminating in a tooth or claw
>palpalis group (subgenus Nemorhina).The outer lateral angle of superior claspers either rounded or strongly produced, not forming a tapering tooth (Figure 1.31c-e)

Outer lateral angles of superior claspers rounded and not strongly produced (Figure 1.31c, d) 14(*palpalis* Group).

Median processes not prominent and generally projecting only slightly if at all between the inferior claspers (Figure 1.33a, d) and never projecting for more than their length (Figure b,c).

Inferior claspers of males of the palpalis group

Inferior claspers with external lobe prominent and projecting at least slightly upwards; internal lobe present and generally prominent (**Figure c-e**) leading to (*fuscipes* s.l.) and the inferior claspers with external lobe not prominent and not projecting, even slightly upward; no internal lobe (**Figure**

a, b) for (p*alpalis* s.l.). The terminal dilation of inferior claspers ("head") in form of a curved pointed hook, the curve prolonging that of the neck; internal lobe of "body" of inferior clasper not projecting strongly (**Figure c**). Central and eastern Africa generally (in region of the great forest and the central African lakes) leading to *fuscipes fuscipes* . The terminal dilation of inferior claspers more or less foot like; internal lobe of inferior claspers may or may not project strongly (**Figure d,e**) .

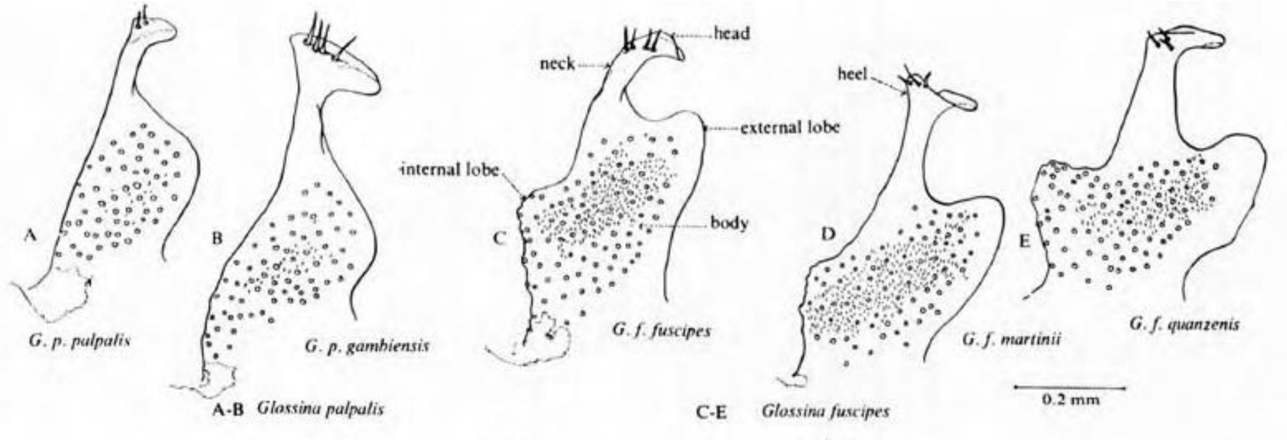

Source: Mulligan 1970

Reference:

Stephen G. A. Leak, Dejene Ejigu, Vreysen, M. J. B. (2008) *Collection of Entomological Baseline Data for Tsetse Area-Wide Integrated Pest Management Programmes*, *FAO*. doi: 10.1046/j.1365-3059.2002.00698.x.
