# Supplementary material for: Genetic diversity and phylogenetic relationships of tsetse flies of the palpalis group in Congo Brazzaville based on mitochondrial cox1 gene sequences
Source: Parasit Vectors. 2020 May 14;13:253. doi: 10.1186/s13071-020-04120-3 (PMC7227191; doi:10.1186/s13071-020-04120-3)
Supplement: Supplementary file 3 — Additional file 3: Figure S1. a Amplification of the cox1 gene for G. fuscipes (s.l.) using the COIF1/COIR1 primer set. Expected amplicon size: 570 bp. b Amplification of cox1 gene for G. p. palpalis using the CI-J-2195/CULR primer set. Expected amplicon size: 850 bp. [file 13071_2020_4120_MOESM3_ESM.docx]

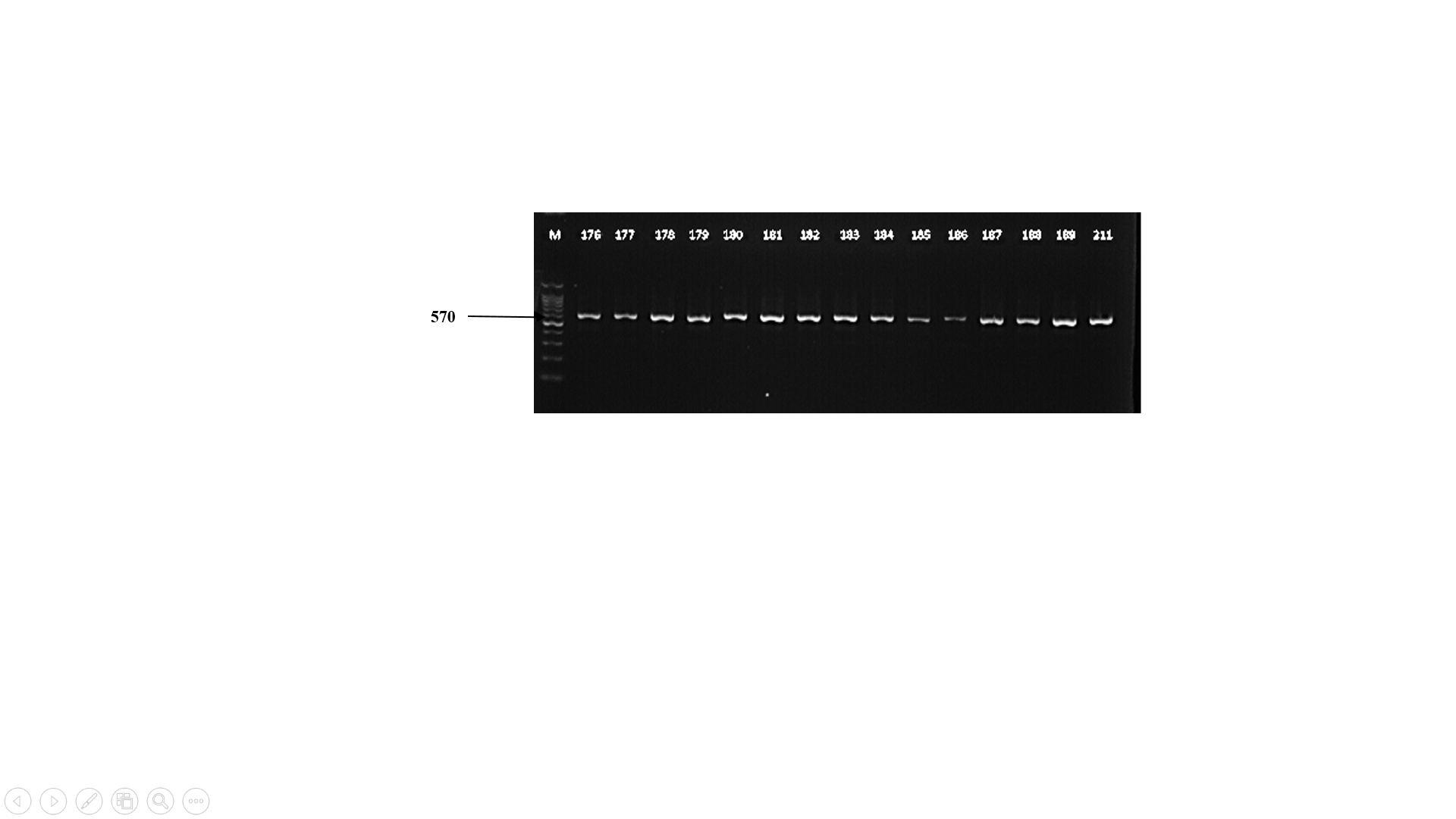


**Additional file 3: Figure S1a**. Amplification of *Glossina fuscipes* (*s.l.*) using COIF1/COIR1 primer set. Expected size is 570bp.


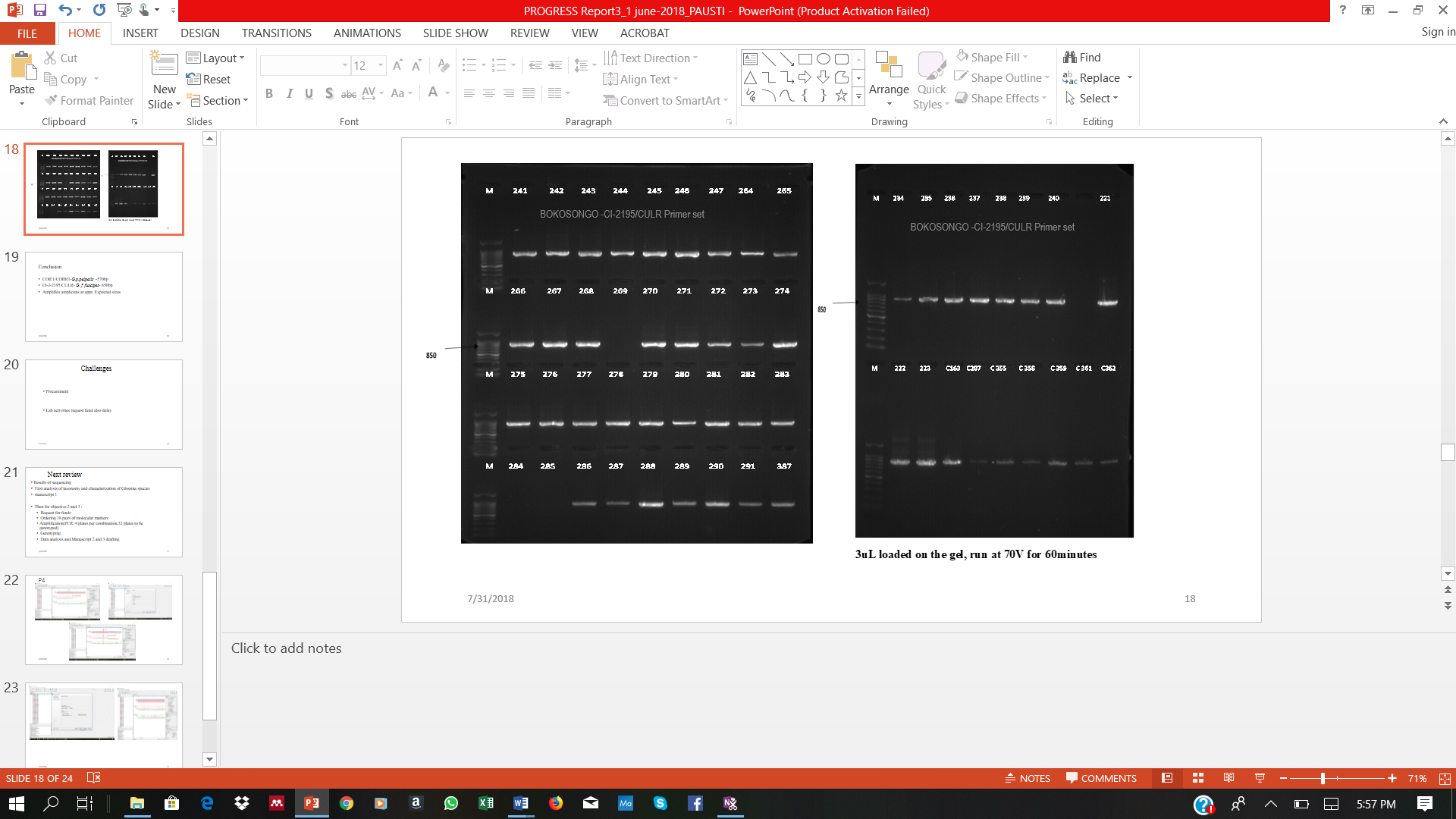


**Additional file 3: Figure S1b**. Amplification of *cox*1 gene in *Glossina palpalis palpalis* using CI-J-2195/CULR primer set. Expected size is 850bp.
