# Supplementary material for: Genetic diversity and phylogenetic relationships of tsetse flies of the palpalis group in Congo Brazzaville based on mitochondrial cox1 gene sequences
Source: Parasit Vectors. 2020 May 14;13:253. doi: 10.1186/s13071-020-04120-3 (PMC7227191; doi:10.1186/s13071-020-04120-3)
Supplement: Supplementary file 4 — Additional file 4: Table S2. Fixation index (FST) based on haplotype frequencies between the three populations of BMSA and TLG and BEMB. [file 13071_2020_4120_MOESM4_ESM.docx]

**Additional file 4: Table S2. Pairwise Fixation index (F_ST_) and other parameters, based on haplotypes frequencies between the three populations, Bomassa (BMSA), Bouemba (BEMB) and Talangai (TLG).**

| POPULATION 1 | POPULATION 2 | **F_ST_** |
| --- | --- | --- |
| BEMB_pop1 | TLG_pop2 | **0.072** |
| BEMB_pop1 | BMSA_pop3 | **0.152** |
| TLG_pop2 | BMSA_pop3 | **0.048** |

F_ST_ (Bomassa Vs Bouemba) = 0.152, p=0.000), p<0.0001); F_ST_ (Bomassa Vs Talangai) = 0.048, p=0.000), p<0.0001)

F_ST_ (Bouemba Vs Talangai) =0.072, P=0.000), p<0.0001).
