# Supplementary material for: Genetic diversity and phylogenetic relationships of tsetse flies of the palpalis group in Congo Brazzaville based on mitochondrial cox1 gene sequences
Source: Parasit Vectors. 2020 May 14;13:253. doi: 10.1186/s13071-020-04120-3 (PMC7227191; doi:10.1186/s13071-020-04120-3)
Supplement: Supplementary file 5 — Additional file 5: Table S3. Pairwise FST values among nine G. f. fuscipes subpopulations from three population groups in Congo Brazzaville. Values written in bold represent significant pairwise differentiation (p < 0.05). FSTP-values, matrix of significant FSTP-values, significance level = 0.05 are highlighted in bold. [file 13071_2020_4120_MOESM5_ESM.docx]

**Additional file 5: Table S3. Matrix of significant F_ST_ p-values. Pairwise F_ST_ values among nine *Gff* subpopulations from three population groups in Congo Brazzaville. Values written in bold represent significant pairwise differentiation (*p <* 0.05). Significance Level=0.05 in bold.**

|  | **BEMB_1** | **BEMB_2** | **BEMB_3** | **TLG_1** | **TLG2** | **TLG 3** | **BMSA_1** | **BMSA_2** | **BMSA_3** |
| --- | --- | --- | --- | --- | --- | --- | --- | --- | --- |
| **BEMB_1** | 0.00000 |  |  |  |  |  |  |  |  |
| **BEMB_2** | -0.01826 | 0.00000 |  |  |  |  |  |  |  |
| **BEMB_3** | -0.01151 | -0.01502 | 0.00000 |  |  |  |  |  |  |
| TLG_1 | **0.046** | **0.053** | **0.059** | 0.00000 |  |  |  |  |  |
| TLG_2 | **0.138** | **0.153** | **0.192** | -0.00795 | 0.00000 |  |  |  |  |
| TLG_3 | 0.01434 | **0.036** | **0.063** | 0.00946 | 0.03168 | 0.00000 |  |  |  |
| BMSA_1 | **0.237** | **0.257** | **0.286** | **0.048** | **0.123** | 0.15242 | 0.00000 |  |  |
| BMSA_2 | **0.076** | **0.084** | **0.094** | **0.001** | **0.037** | **0.045** | 0.00934 | 0.00000 |  |
| BMSA_3 | **0.194** | **0.214** | **0.242** | **0.034** | **0.096** | **0.118** | -0.00616 | -0.00611 | 0.00000 |
