# Supplementary material for: Genetic diversity and phylogenetic relationships of tsetse flies of the palpalis group in Congo Brazzaville based on mitochondrial cox1 gene sequences
Source: Parasit Vectors. 2020 May 14;13:253. doi: 10.1186/s13071-020-04120-3 (PMC7227191; doi:10.1186/s13071-020-04120-3)
Supplement: Supplementary file 6 — Additional file 6: Table S4. Hierarchical analysis of molecular variance (AMOVA) design and results based on distance method of genetic structure [40]. Pairwise difference based on the two ecological localities, Plateau Batéké (BEMB-TLG) against (BMSA) rainforest, to test whether the genetic differentiation is influenced by ecology or environmental conditions/factors. [file 13071_2020_4120_MOESM6_ESM.docx]

**Additional file 6: Table S4.** Hierarchical analysis of molecular variance (AMOVA) design and results based on Distance method of Genetic structure [41]. Pairwise difference based on the 2 ecological localities Plateau Batéké (BEMB-TLG) against (BMSA) Rainforest, to test whether the genetic differentiation is influenced by ecology or environmental conditions/factors.

| **Source of Variation** | **d.f** | **Sum of squares** | **Variance components** | **Percentage of variation** | **Fixation Index (P-value)** |
| --- | --- | --- | --- | --- | --- |
| Among groups | 1 | 30.000 | 0.21880 Va | 7.93% | F_CT_: 0.07926 (0.00880) |
| Among subpopulations  within groups | 7 | 30.582 | 0.06478 Vb | 2.35% | F_SC_ : 0.02549 (0.00000) |
| Among individuals within populations | 254 | 629.160 | 2.47701 Vc | 89.73% | F_ST_ :0.10273 (0.00880) |
| Total | 262 | 689.741 |  |  |  |

Abbreviations: Va, Variance of a; Vb, Variance of b; Vc: Variance of c. Fixation Index.
