# Supplementary figures and images for: Genetic diversity and phylogenetic relationships of tsetse flies of the palpalis group in Congo Brazzaville based on mitochondrial cox1 gene sequences
Source: Parasit Vectors. 2020 May 14;13:253. doi: 10.1186/s13071-020-04120-3 (PMC7227191; doi:10.1186/s13071-020-04120-3)

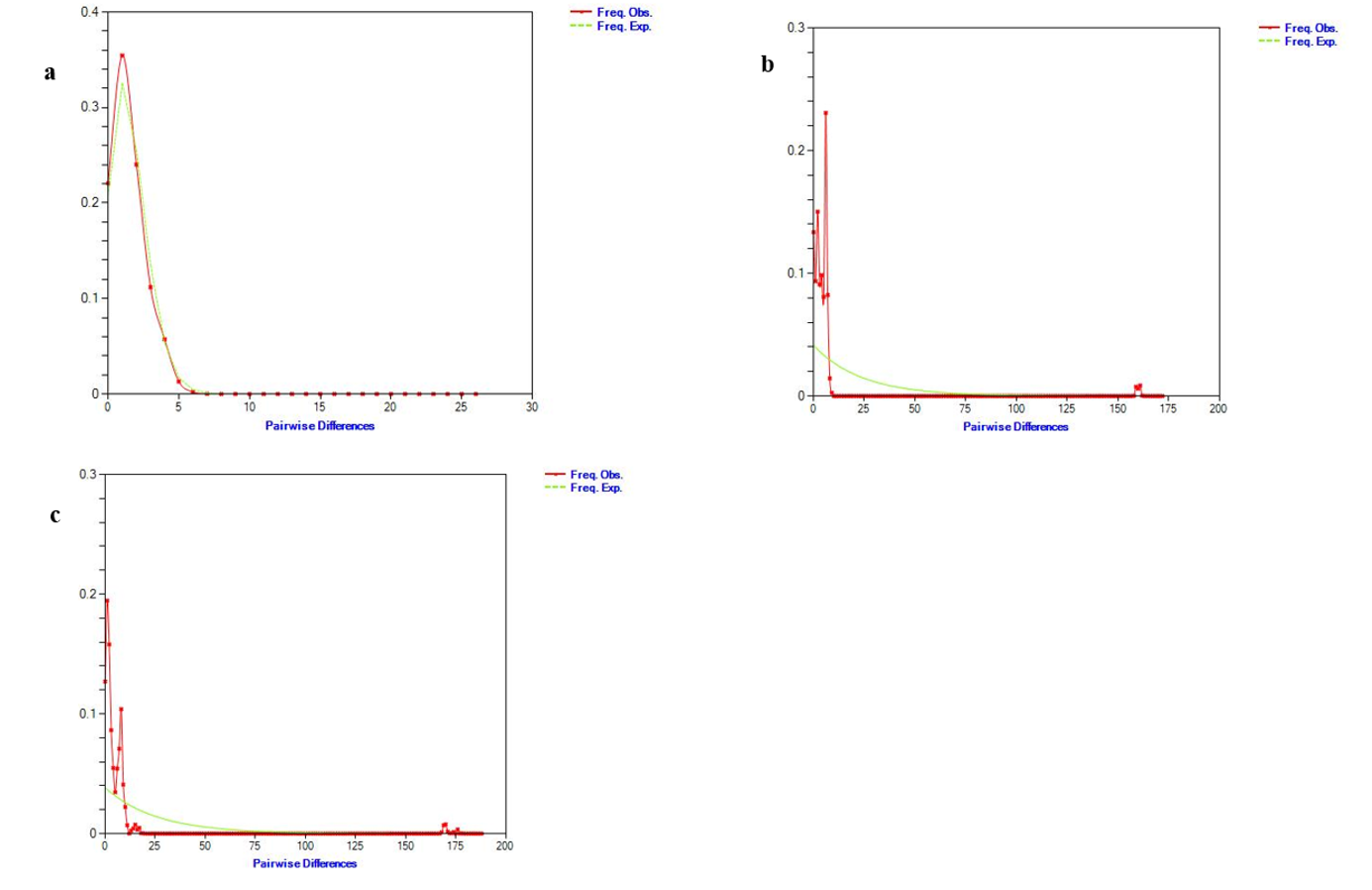

Supplement: Supplementary file 7 — Additional file 7: Figure S2. Mismatch distribution of haplotype pairwise nucleotide differences for G. fuscipes (s.l.) in the three localities, BEMB (a), TLG (b) and BMSA (c), showing observed (red lines) and expected (green lines) frequencies obtained under a model following populations’ size change. [file 13071_2020_4120_MOESM7_ESM.tif]
