# Supplementary material for: Genetic diversity and phylogenetic relationships of tsetse flies of the palpalis group in Congo Brazzaville based on mitochondrial cox1 gene sequences
Source: Parasit Vectors. 2020 May 14;13:253. doi: 10.1186/s13071-020-04120-3 (PMC7227191; doi:10.1186/s13071-020-04120-3)
Supplement: Supplementary file 8 — Additional file 8: Table S5. Mitochondrial cox1 gene haplotype IDs, frequencies and order positions for G. fuscipes (s.l.) from Congo Brazzaville. [file 13071_2020_4120_MOESM8_ESM.docx]

**Additional file 8: Table S5*.*** Mitochondrial *cox*1 gene haplotype IDs, frequencies and order positions for *G. fuscipes* (*s.l.*) from Congo Brazzaville.

| **Haplotype ID** | **Haplotype frequency#** | **Haplotype Individual sequences** | **Haplotype Order # and position** |
| --- | --- | --- | --- |
| Hap_1 | 61 | BEMB161 BEMB162 BEMB165 BEMB168 BEMB170 BEMB171 BEMB172 BEMB175 BEMB176 BEMB178 BEMB179 BEMB183 BEMB188 BEMB190 BEMB191 BEMB192 BEMB194 BEMB197 BEMB199 BEMB200 BEMB202 BEMB203 BEMB205 BEMB206 BEMB211 BEMB212 BEMB213 BEMB214 BEMB216 BEMB218 BEMB221 BEMB222 BEMB223 BEMB195 BEMB224 BEMB226 BEMB228 BEMB230 BEMB232 BEMB233 BEMB388 BEMB389 BEMB390 BEMB392 BEMB393 BEMB394 BEMB397 BEMB398 BEMB399 BEMB401 BEMB403 BEMB423 BEMB425 BEMB426BMSA21 TLG441 TLG442 TLG449 TLG450 TLG453 TLG459 | [1-2 4 7 9-11 14-17 21 26 28-30 32 34 36-39 41-42 47-50 52 54 57-62 64 66 68-72 74-76 79-82 84-85 87-88 102 237-238 256-257 260 263] |
| Hap_2 | 2 | BEMB164TLG436 | [3 235] |
| Hap_3 | 54 | BEMB166 BEMB169 BEMB174 BEMB180 BEMB182 BEMB187 BEMB189 BEMB193 BEMB196 BEMB210 BEMB215 BEMB220 BEMB227 BEMB229 BEMB231 BEMB396 BEMB402 BEMB424BMSA70TLG100 TLG101 TLG121 TLG125 TLG128 TLG87 TLG89 TLG90 TLG91 TLG97 TLG98 TLG99 TLG111 TLG112 TLG114 TLG117 TLG118 TLG136 TLG137 TLG138 TLG139 TLG140 TLG144 TLG145 TLG146 TLG147 TLG149 TLG150 TLG155 TLG156 TLG158 TLG159 TLG446 TLG447 TLG454 | [5 8 13 18 20 25 27 31 33 46 51 56 63 65 67 78 83 86 140 177-179 182 185 192 194-196 201-203 211-212 214 217-218 222-226 230-234 240 243-244 246-247 254-255 261] |
| Hap_4 | 20 | BEMB167 BEMB204 BMSA12 BMSA13 BMSA20 BMSA58 BMSA6 BMSA63 BMSA64 BMSA67 BMSA68 BMSA72 BMSA74 BMSA358 BMSA393 BMSA43 BMSA46 BMSA80 BMSA83 BMSA85] | [6 40 92-93 101 126 128 132-133 136-137 142 144 155 160 164 166 171 174 176] |
| Hap_5 | 6 | BEMB173 BEMB208 BEMB209 BEMB217 BEMB395TLG439 | [12 44-45 53 77 250] |
| Hap_6 | 8 | BEMB181 BEMB184 BEMB185 BEMB198TLG443 TLG440 TLG445 TLG455 | [19 22-23 35 239 251 253 262] |
| Hap_7 | 1 | BEMB186 | [24] |
| Hap_8 | 1 | BEMB207 | [43] |
| Hap_9 | 2 | BEMB219 BEMB391 | [55 73] |
| Hap_10 | 12 | BMSA1 BMSA11 BMSA14 BMSA22 BMSA25 BMSA49 BMSA5 BMSA50 BMSA55 BMSA56 BMSA9 BMSA47] | [89 91 94 103 106 116-118 123-124 148 167] |
| Hap_11 | 25 | BMSA10 BMSA16 BMSA17 BMSA18 BMSA19 BMSA23 BMSA26 BMSA29 BMSA3 BMSA32 BMSA52 BMSA53 BMSA54 BMSA62 BMSA66 BMSA73 BMSA76 BMSA8 BMSA355 BMSA356 BMSA36 BMSA39 BMSA45 BMSA82 BMSA84] | [90 96-99 104 107 110-111 113 120-122 131 135 143 146-147 152-153 156 159 165 173 175] |
| Hap_12 | 1 | BMSA15] | [95] |
| Hap_13 | 2 | BMSA2 BMSA7] | [100 139] |
| Hap_14 | 3 | BMSA24 BMSA357 BMSA42] | [105 154 163] |
| Hap_15 | 1 | BMSA27] | [108] |
| Hap_16 | 1 | BMSA28] | [109] |
| Hap_17 | 3 | BMSA31 BMSA40 BMSA78] | [112 161 169] |
| Hap_18 | 4 | BMSA4 BMSA61 BMSA69 BMSA34] | [114 130 138 150] |
| Hap_19 | 2 | BMSA48 BMSA81] | [115 172] |
| Hap_20 | 2 | BMSA51 TLG107] | [119 208] |
| Hap_21 | 1 | BMSA57] | [125] |
| Hap_22 | 2 | BMSA59 BMSA71] | [127 141] |
| Hap_23 | 1 | BMSA60] | [129] |
| Hap_24 | 1 | BMSA65] | [134] |
| Hap_25 | 1 | BMSA75] | [145] |
| Hap_26 | 2 | BMSA33 BMSA37] | [149 157] |
| Hap_27 | 1 | BMSA35] | [151] |
| Hap_28 | 1 | BMSA38] | [158] |
| Hap_29 | 1 | BMSA41] | [162] |
| Hap_30 | 1 | BMSA77] | [168] |
| Hap_31 | 1 | BMSA79] | [170] |
| Hap_32 | 4 | TLG122 TLG127 TLG94 TLG452] | [180 184 198 259] |
| Hap_33 | 13 | TLG124 TLG129 TLG132 TLG134 TLG88 TLG105 TLG108 TLG109 TLG119 TLG120 TLG142 TLG153 TLG157 | [181 186 189 191 193 206 209-210 219-220 228 242 245] |
| Hap_34 | 5 | TLG126 TLG92 TLG113 TLG135 TLG143] | [183 197 213 221 229] |
| Hap_35 | 6 | TLG130 TLG102 TLG116 TLG141 TLG437 TLG160] | [187 204 216 227 236 248] |
| Hap_36 | 1 | TLG131] | [188] |
| Hap_37 | 1 | TLG133] | [190] |
| Hap_38 | 1 | TLG95 | [199] |
| Hap_39 | 1 | TLG96] | [200] |
| Hap_40 | 1 | TLG103] | [205] |
| Hap_41 | 1 | TLG106] | [207] |
| Hap_42 | 1 | TLG115] | [215] |
| Hap_43 | 1 | [TLG151] | [241] |
| Hap_44 | 1 | TLG438] | [249] |
| Hap_45 | 1 | TLG444] | [252] |
| Hap_46 | 1 | TLG451] | [258] |
